# Exosome‐transmitted linc00852 associated with receptor tyrosine kinase AXL dysregulates the proliferation and invasion of osteosarcoma

**DOI:** 10.1002/cam4.3303

**Published:** 2020-07-16

**Authors:** Qiming Li, Xuedi Wang, Nian Jiang, Xianbiao Xie, Ni Liu, JunFeng Liu, Jingnan Shen, Tingsheng Peng

**Affiliations:** ^1^ Department of Pathology First Affiliated Hospital of Sun Yat‐sen University Guangzhou P. R. China; ^2^ Department of Musculoskeletal Oncology First Affiliated Hospital of Sun Yat‐sen University Guangzhou P. R. China

**Keywords:** AXL, exosome, invasion, linc00852, osteosarcoma

## Abstract

**Background:**

Receptor tyrosine kinase AXL has been found to be highly expressed in osteosarcoma and positively associated with poor prognosis. There are tumor groups with high or low AXL expression, which had different capabilities of invading vessels and forming distal metastases. Exosome‐transmitted lncRNA may be transferred intercellularly to promote tumor cells’ proliferation and invasion.

**Methods:**

Exosomes were detected by electron microscopy, particle size analysis, and western blotting. High‐throughput sequencing helped to find the highest differentially expressed lncRNA in AXL‐associated exosomes. Clone formation, wound healing, transwell assay, and xenograft model in nude mice were performed to evaluate cells’ proliferation, migration, and invasion in vitro and in vivo. Lentiviral transfection was used to up‐ or down‐regulate the lncRNA levels in cell lines. Luciferase reporter assay and RNA FISH etchelped to indicate the molecular mechanisms. The results in the cell lines were proved in the osteosarcoma tissues with clinical analysis.

**Results:**

The exosomes derived from donor cells with high AXL expression could promote the proliferation and invasion and upregulate AXL expression of the receiver cells with low AXL. Linc00852 was the highest differentially expressed lncRNA in AXL‐associated exosomes and was also regulated by AXL expression. Although the mechanisms of linc00852 in nucleus were unrevealed, it could upregulate AXL expression partly by competitively binding to miR‐7‐5p. The AXL‐exosome‐linc00852‐AXL positive feedback loop might exist between the donor cells and the receiver cells. Clinically, linc00852 was significantly highly expressed in osteosarcoma tissues and positively associated with tumor volumes and metastases, which was also obviously related with AXL mRNA expression.

**Conclusion:**

AXL‐associated exosomal linc00852 up‐regulated the proliferation, migration, and invasion of osteosarcoma cells, which would be considered as a new tumor biomarker and a special therapeutic target for osteosarcoma.

## Graphical abstract

Exosomal linc00852 promotes osteosarcoma migration and invasion by upregulating AXL. AXL‐associated exosomal linc00852 may be considered as a new tumor biomarker and a special therapeutic target for osteosarcoma.

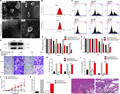

**Lay summary**

AXL has been proven to be important in osteosarcoma and there are tumor groups with high or low AXL expression. The present study shows exosomes derived from donor cells with high AXL expression could be transmitted to receiver cells with low AXL, and the exosomes transferred intercellularly promoted tumor cells proliferation and invasion. linc00852 was the highest differentially expressed lncRNA in AXL‐associated exosomes, which was demonstrated to upregulate AXL expression and promote cells progression. AXL‐associated exosomal linc00852 may be considered as a special therapeutic target and a new tumor biomarker for osteosarcoma.

## 1 INTRODUCTION

Osteosarcoma is a common primary malignant bone tumor, which occurs mainly in children and adolescents, comprising almost 60% of all sarcoma cases.^1^ It occurs mostly in the metaphysis of long bones and grows rapidly with strong ability of invasion and destruction. The prognosis of the patients with metastatic lesions is much worse than the patients only with primary tumors.^2^ Exploring the underlying mechanism of osteosarcoma invasion and metastasis is helpful for understanding the occurrence and development of osteosarcoma.

The AXL receptor tyrosine kinase (AXL) is a founding member of TAM (Tyro3‐AXL‐Mer) family, which has high affinity with the ligand as growth arrest specific protein 6 (Gas6).^3^ The Gas6/AXL signaling pathway is related to tumor cell growth, invasion, metastasis, epithelial mesenchymal transition (EMT), angiogenesis, and so forth.^4^, ^5^, ^6^ AXL was highly expressed in osteosarcoma and positively associated with a poor prognosis.^1^, ^7^, ^8^ We found that the positive rate and the intensity of the P‐AXL expression were much higher in the tumor cells with obvious atypia than that with mild morphology, or in the perivascular tumor cells than that far away from the blood vessels (Figure S1). There were different tumor groups with different levels of AXL in osteosarcoma, which had different capabilities of invading vessels and forming distal metastases. According to the theory of tumor heterogeneity, tumor is not an isolated entity. The interaction between tumor cells and adjacent cells in primary tumor is the key to tumor development, and the intercellular communication also carries out through a complex system containing secretory factors, which may play central role in the interaction among cells located far away from each other.^9^ With the understanding of exosomes, we hypothesized that exosomes would be the important secreted factor in the communication between the two groups with high and low AXL expression in osteosarcoma.

Exosomes are small extracellular vesicles (EVs) discovered in 1983 and ranging in size from 40 to 150 nm. They originate from the multicystic endosomes and are actively secreted by all types of cells after fusion of the multicysts and plasmalemma. Exosomes are packed with lipids, proteins, DNAs, mRNAs, lncRNAs, and miRNAs, representing a new way of intracellular communication.^9^, ^10^, ^11^, ^12^, ^13^ Cancer‐derived exosomes have the ability to promote tumor invasion and metastasis potential to the recipient cells.^12^, ^14^ Nevertheless, the roles of lncRNAs transferred by exosomes between different AXL levels in osteosarcoma are poorly understood. In this study, we investigated the contributions of lncRNA transferred by exosomes from donor cells with high AXL expression to receiver cells with low AXL during the process of the invasion and metastasis of osteosarcoma, and attempted to reveal the mechanisms.

## 2 RESULTS

### 2.1 Identification of exosomes secreted by osteosarcoma cells with different AXL expression levels

**FIGURE 1:**
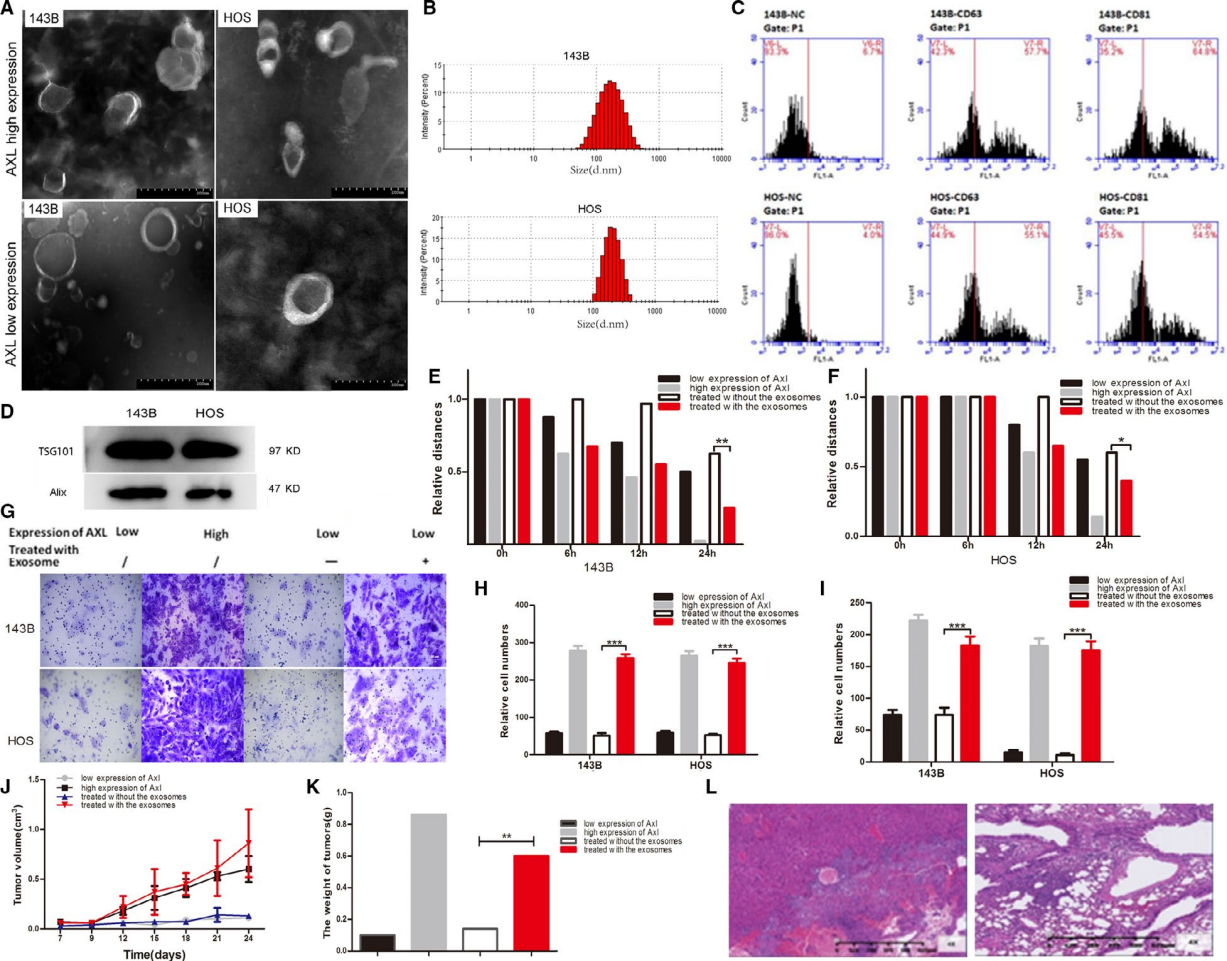
Characterization of exosomes derived from osteosarcoma cells with high AXL expression and the functions of the exosomes after being transmitted to receiver cells with low AXL expression. A, Electron microscopy images of exosomes derived from osteosarcoma cells 143B and HOS with high and low AXL expression. B, The size distribution of exosomes derived from 143B and HOS were detected by a particle size analysis; the particle size ranged from 40 to 200 nm, accounting for 51.3% and 67.1%, respectively. C, Exosomes derived from 143B and HOS cells were analyzed by flow cytometry with CD63 and CD81. D, Exosomes were analyzed by Western blot with TSG101 and Alix. E, Wound healing assays showed that the migration ability of 143B cells with low AXL expression increased significantly after co‐culturing with the exosomes derived from cells with high AXL expression for 24 h. F, Similarly, the exosomes also increased the migration ability of HOS. G,H, The Transwell assays showed the migration abilities of 143B and HOS. The number of migrated cells with high AXL expression was significantly higher than the number of migrated cells with low AXL expression. Meanwhile, the number of the migrated cells with low AXL expression increased significantly after 24 h of co‐culturing with the exosomes derived from the cells with high AXL expression. I, The Matrigel‐coated Boyden chamber assays showed the invasive ability of 143B and HOS cells with low AXL expression similarly increased significantly with treatment of the exosomes. J, The growth curves of 143B with different AXL levels in nude mice. The xenograft tumors with high AXL expression grew faster than the tumors with low AXL expression. After treating with the exosomes derived from the cells with high AXL expression, the growth rate of the xenograft tumors with low AXL expression increased significantly. K, The weight of xenograft tumors with high AXL expression was greater than that of the tumors with low AXL expression. The weight of xenograft tumors of cells co‐cultured with the exosomes increased significantly the weight of tumors with low AXL expression. L, H&E staining showed that the pathological features of xenograft tumors were similar to the ordinary osteosarcoma. One lung metastatic foci occurred in the group of cells co‐cultured with the exosomes compared with no metastasis of the untreated xenograft tumors. (*P < .05, **P < .01, ***P < .001)

Osteosarcoma cell‐derived exosomes with high or low AXL expression were isolated from the conditioned medium of 143B and HOS cells, which were sorted and recovered by flow cytometry. Transmission electron microscopy (TEM) was used to analyze the morphology of exosomes. The exosomes with different AXL expression levels showed the nanoscale particle morphology with lipid bilayer‐coated, the size of these particles were 40‐200 nm (Figure 1A,B). CD63 and CD81 were detected in the exosomes produced from osteosarcoma cells by flow cytometry. The positive rates of CD63 and CD81 were 55.1% and 54.5% in HOS, while they were 57.7% and 64.8% in 143B separately (Figure 1C). Two protein markers in the exosomes from 143B and HOS as TSG101 and Alix were detected by Western blots (Figure 1D). These results indicated that osteosarcoma cell lines with high or low AXL expression could secrete exosomes with common exosomal features.

### 2.2 Exosomes derived from osteosarcoma cells with AXL high expression promoted the proliferation, migration and invasion of the cells with low AXL

In both 143B and HOS, the cells co‐cultured with the exosomes derived from high AXL expression cells had stronger ability for migration and invasion compared to the untreated cells with low AXL expression. In the migration assay, the distance of the scratch were measured at 0h, 12h, 24h, then the average relative distance at different point were gained when they were relative to the distance at 0h. When 143B cells with low AXL expression were co‐cultured with the exosomes for 24 hours, the average relative distance decreased from 0.625 to 0.25 (Figure 1E, Figure S2; *P* = .003). Similarly, the average relative distance decreased from 0.6 to 0.4 for HOS cells treated with the exosomes (Figure 1F, Figure S3; *P* = .029). In the invasion assays, the average number of 143B cells which passed through the Transwell microporous membrane increased from 51 to 259, while that of HOS increased from 52 to 246 (Figure 1G,H; *P* = .000). Similarly, the average number of 143B cells passed through the matrigel‐coated Boyden chamber microporous membrane increased from 74 to 183, while that of HOS increased from 11 to 175 (Figure 1I, Fig. S4; *P* = .000).These results indicated that the exosomes from osteosarcoma cells with high AXL expression could increase migration and invasion of osteosarcoma cells with low AXL in vitro. Comparing to the untreated 143B with low AXL expression, the growth rate of the xenograft tumors of 143B pre‐treated with the exosomes from the cells with high AXL increased dramatically in nude mice (Figure 1J; *P* = .004).The weight of the xenograft tumors of pretreated 143B also increased obviously than the cell untreated (Figure 1K, Figure S5; *P* < .01). These results proved that the special exosomes could also enhance the growth and invasion of osteosarcoma cell 143B in vivo. One case in the treated group had liver and lung metastatic foci (Figure 1L), whereas there were no metastatic foci in the group with low AXL expression.

### 2.3 Linc00852 was identified as the highest differentially expressed lncRNA in AXL‐associated exsomes and was upregulated by AXL

**FIGURE 2:**
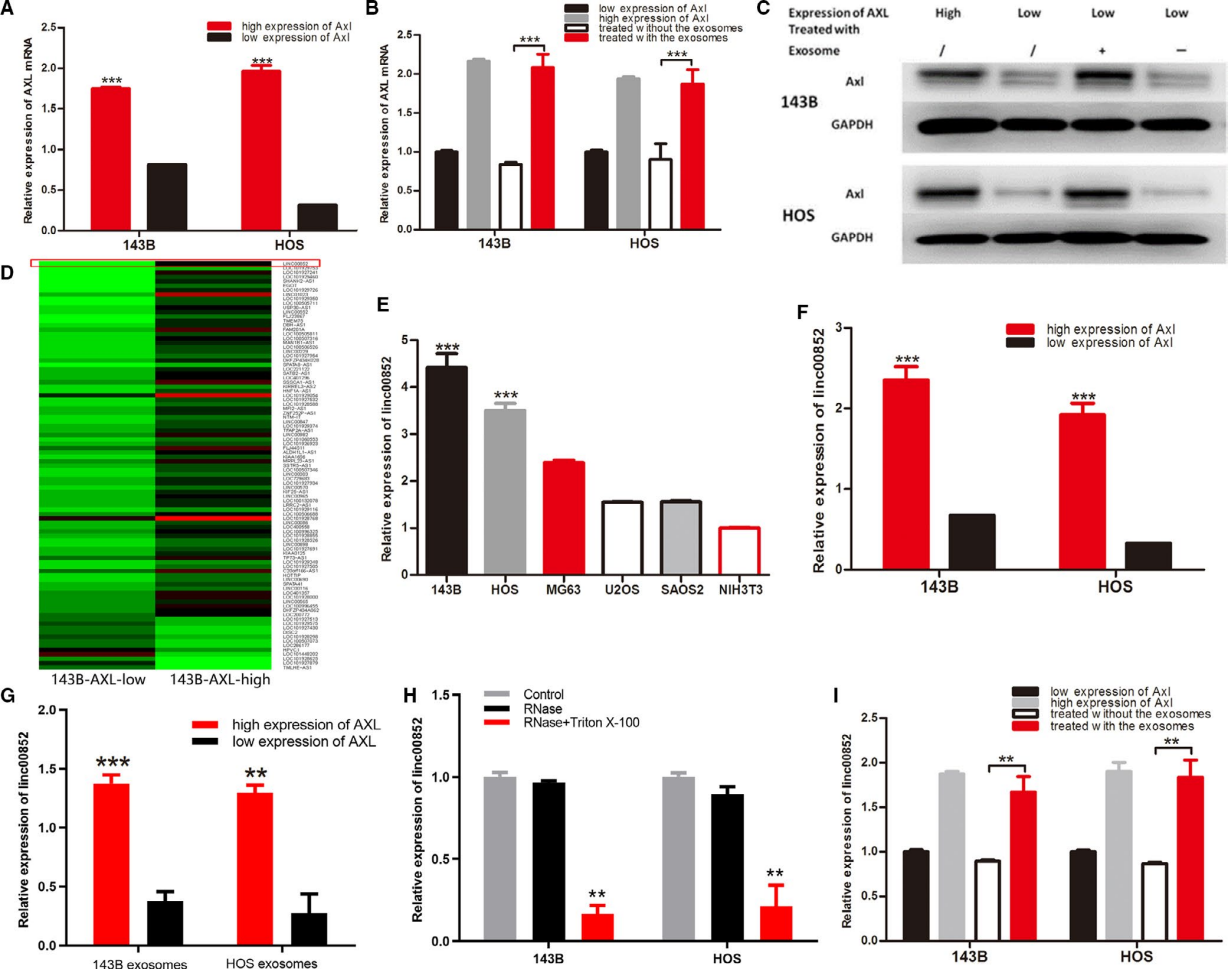
Exosomes derived from donor cells with high AXL expression promoted the expression of AXL mRNA and protein in receiver cells with low AXL expression, and linc00852 was the highest differentially expressed lncRNA in AXL‐associated exosomes. A, AXL mRNA expression by qRT‐PCR detection was significantly higher in cells with high AXL expression than in cells with low AXL expression by flow cytometry for both 143B and HOS. B, The AXL mRNA expression of groups with low AXL expression increased significantly both for 143B and HOS after cells with low AXL expression were co‐cultured with exosomes derived from cells with high AXL expression 24 h. C, AXL protein expression was similar to mRNA by Western blot. D, High‐throughput sequencing analysis revealed that there were many lncRNAs increased in the exosomes from 143B with highly expressed AXL (right) compared with the exosomes from 143B with AXL low expression (left), among which linc00852 was the highest differentially expressed lncRNA. E, The linc00852 expression by qRT‐PCR detection in osteosarcoma cell lines was significantly higher than that in NIH3T3 cells, and linc00852 was detected at relatively high levels in 143B and HOS. F, The expression of linc00852 in the cells with high AXL expression was significantly higher than in the cells with low AXL expression. G, The expression of linc00852 in exosomes derived from cells with high AXL expression was significantly higher than in cells with low AXL expression. H, The expression of linc00852 in the exosomes decreased dramatically after being treated with 2 μg/ml RNase and Triton 0.1%X‐100, whereas there was no obvious change with the treatment of RNase alone in both 143B and HOS cells. I, The linc00852 expression of the cells with low AXL expression increased significantly both for 143B and HOS after they were co‐cultured with exosomes derived from cells with high AXL expression for 24 h. (*P < .05, **P < .01, ***P < .001)

Both in 143B and HOS, AXL mRNA was consistent with the protein in different AXL expression groups sorted by the flow cytometry assays (Figure 2A 143B; *P* = .000 HOS; *P* = .000). After the cells with low AXL were treated with the exosomes derived from the cells with high AXL for 24hrs, AXL mRNA increased nearly double times(Figure 2B 143B; *P* < .001, HOS; *P* < .001). And the expression of AXL protein also increased obviously (Figure 2C 143B; *P* = .000, HOS; *P* = .004). To identify the difference of lncRNAs in the exosomes derived from the cell with high AXL expression and that from the cell with low AXL, high‐throughput sequencing were repeated twice in osteosarcoma cell 143B. As shown in the sequencing map, there were many lncRNAs highly expressed in the exosomes of 143B with high AXL expression compared to that of 143B with low AXL, among which linc00852 was the highest differentially expressed lncRNA (Figure 2D; *P* = .000). Then, linc00852 was confirmed by qRT‐PCR to be highly expressed in osteosarcoma cell lines as 143B, HOS, MG63, U2OS, and SAOS2, which were significantly higher than that in the NIH3T3 cells. Furthermore, linc00852 was especially highly expressed in the poorly differentiated osteosarcoma cell lines as 143B and HOS, which were 3.4 and 2.5 times higher than that in NIH3T3 cells, separately(Figure 2E 143B; *P* = .000, HOS; *P* = .000). On the other hand, the expression of linc00852 was associated with the expression of AXL. Both in HOS and 143B, linc00852 in the cells with high AXL expression was 3.5 and 5.8 times than that in the cells with low AXL (Figure 2F 143B; *P* = .000, HOS; *P* = .000). Consistently, linc00852 was also expressed obviously higher in the exosomes from the cells with high AXL than that from the cells with low AXL (Figure 2G 143B; *P* = .000 HOS; *P* = .001). After RNase treatment, the concentration of linc00852 in the condition medium of both 143B and HOS did not change obviously, while it was obviously reduced when treated concurrently with RNase and Triton X‐100 (Figure 2H; *P < *.01). Those results proved that extracellular linc00852 was mainly enveloped by exosomes rather than released directly. Similar to the expression of AXL mRNA, linc00852 was consistent with the concentration of AXL protein expression. And linc00852 also increased significantly with the treatment of the exosomes derived from the cells with high AXL in both 143B and HOS (Figure 2I 143B; *P* = .001, HOS; *P* = .001).

### 2.4 Linc00852 promoted osteosarcoma cell proliferation, migration, and invasion in vitro and in vivo

**FIGURE 3:**
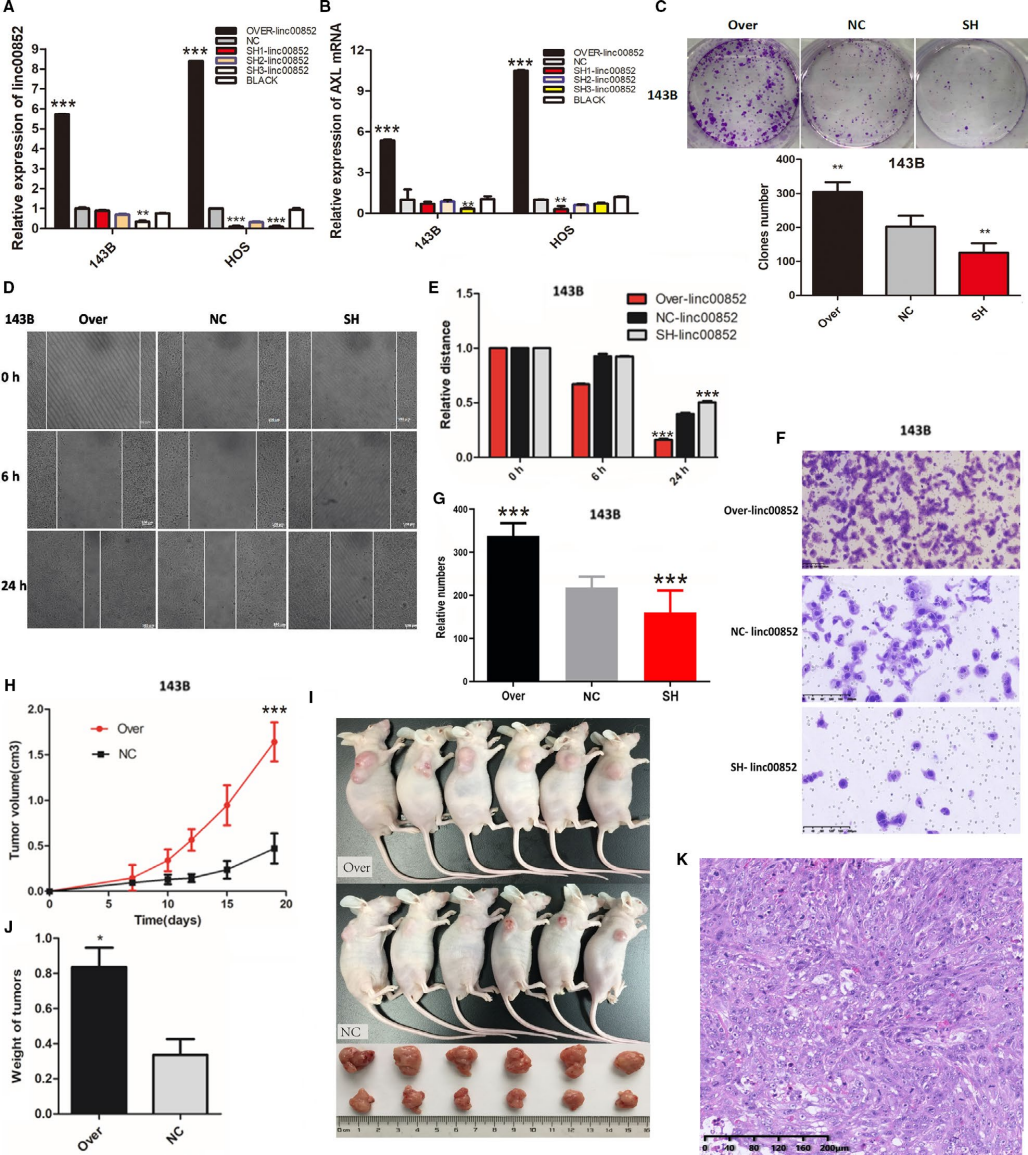
Exosomal linc00852 promoted proliferation, migration and invasion of osteosarcoma cells in vitro and in vivo. A, The transfection efficiency was detected by qRT‐PCR. Linc00852 was highly expressed in both 143B and HOS cells transfected with lentiviral vectors encoding the full linc00852 sequence. Data are shown as the mean ± SD. B, AXL mRNA expression of transfected 143B and HOS cells were detected by qRT‐PCR. The trends of AXL expression were similar to the linc00852 expression. C, Clone formation assays showed that in 143B the clone numbers were increased significantly in over‐linc00852 cells and decreased significantly in SH‐linc00852 cells compared with the control. D, The migration ability of 143B cells was detected with wound healing assays. E, Compared with the control, the migration distance of over‐linc00852 cells significantly decreased, and the migration distance of SH‐linc00852 cells significantly increased after 24 h. F, The invasive potential of 143B cells was detected by Matrigel‐coated Transwell assays. G, Compared with the control, the invasive ability of over‐linc00852 cells was significantly enhanced. H, The growth curve of transfected 143B cells in nude mice. Over‐linc00852 xenograft tumors grew significantly faster than the NC‐linc00852 group. I, The size of the xenograft tumors in the over‐linc00852 group was significantly larger than in the control group. J, The weight of the xenograft tumors in the over‐linc00852 group was significantly greater than in the control group. K, H&E staining showed that the pathological features of the xenograft tumors were similar to ordinary osteosarcoma. (*P < .05, **P < .01, ***P < .001)

To investigate the exact function of linc00852 in osteosarcoma cells, we ectopically increased the linc00852 level using lentiviral vectors in 143B and HOS cells and suppressed linc00852 expression using lentiviral shRNAs. The plasmid map of the lentiviral vector (GV367) encoding the full linc00852 sequence was shown (Figure S6). Cells transfected with scramble vector and empty vector were set as the negative controls. Compared with the negative control cells (Figure S7,S9), linc00852 increased significantly both in 143B and HOS transfected with lentiviral plasmid encoding the full linc00852 sequence (Figure S8, S10). The expression of linc00852 was almost six times higher in 143B and almost nine times higher in HOS than that in the control group（143B *P* = .000; HOS *P* = .000）. Whereas linc00852 decreased dramatically in the cells transfected with lentiviral shRNAs, and there was a threefold reduction and a 100‐fold reduction of linc00852 expression in 143B and HOS cells respectively (143B *P* = .003; HOS *P* = .000) (Figure 3A). In consistent with the expression of linc00852, AXL mRNA expression increased with 5.2 and 10.7 times enhancement in 143B and HOS cells transfected with lentiviral vectors (Figure 3B 143B; *P* = .000; HOS; *P* = .000). Compared with the control cells, the clone numbers significantly increased from 203 to 291 in cells with overexpressed linc00852, but obviously decreased from 203 to 125 in cells with linc00852 knockdown, which proved that linc00852 overexpression significantly increased the proliferation of 143B cells (Figure 3C; *P* = .005). Compared with the control group, the relative distance of overexpressed linc00852 decreased from 0.398 to 0.163 after 24 hours, indicating that linc00852 overexpression significantly increased the migration of 143B cells (Figure 3D,E; *P* = .000). Compared with the control group, the average number of the cells which passed through the Transwell microporous membrane in linc00852 overexpressed group increased from 223 to 325, which in the linc00852 knockdown group decreased from 223 to 151 after 48 hours (Figure 3F,G; *P* = .000). Those results revealed that linc00852 could increase the ability of osteosarcoma cells' invasion in vitro. As shown by the growth curve of the xenograft tumors, the tumor volume of linc00852 overexpressed group was three times bigger than that of the control group, which indicated that linc00852 overexpression also increased the osteosarcoma cells’ growth and invasion in vivo (Figure 3H,I; *P* = .000). The average weight of the xenograft tumors of the control group was 0.336g, which increased to 0.838g in linc00852 overexpression group (Figure 3J; *P* = .000). The xenograft tumor had similar atypical morphology with the osteosarcoma tissues from the patients (Figure 3K).

### 2.5 Linc00852 functioned as a ceRNA for miR‐7‐5p to promote AXL expression in osteosarcoma cell cytoplasm

**FIGURE 4:**
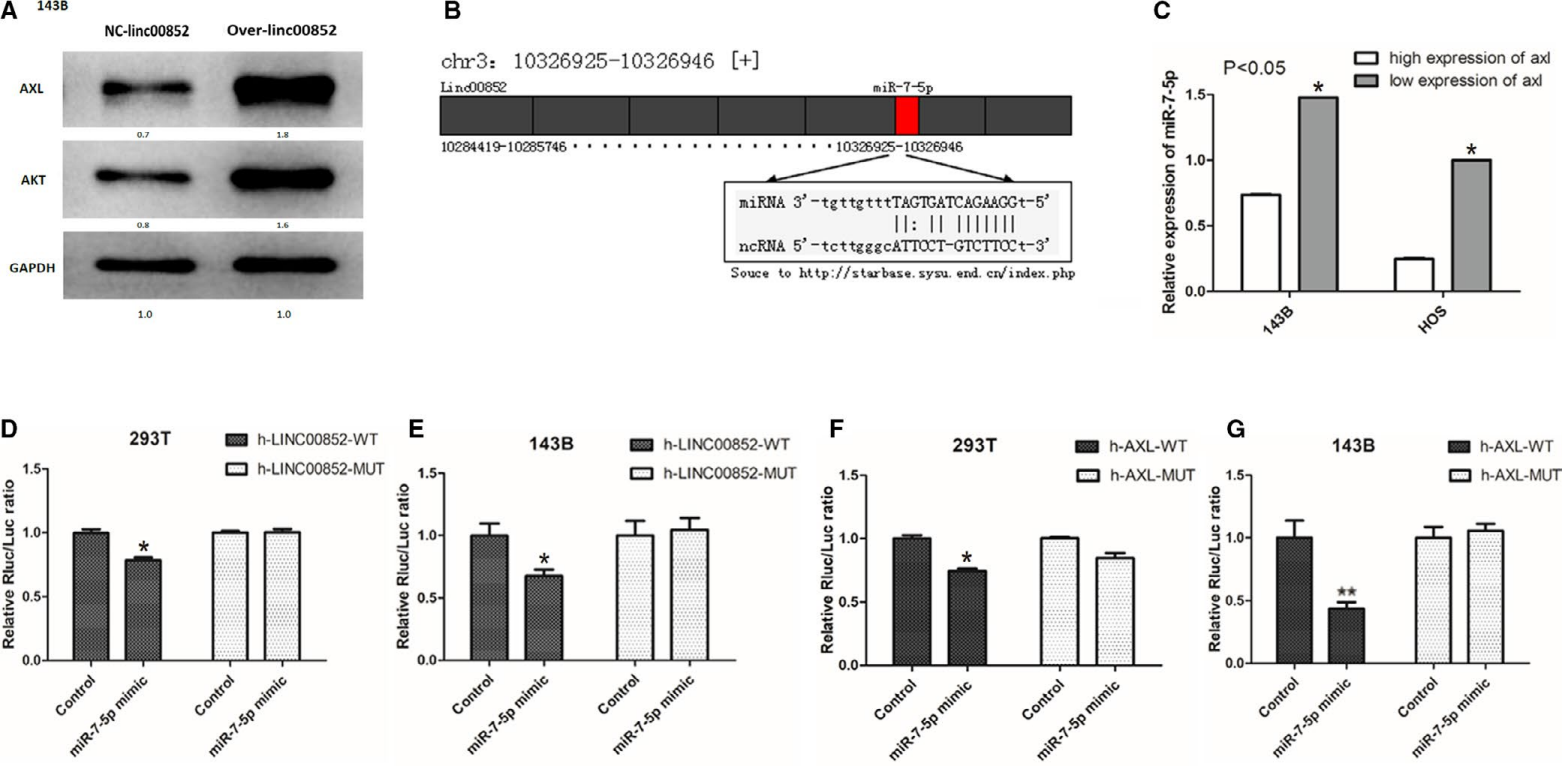
Over‐linc00852 increased the expression of AXL and AKT protein, and linc00852 upregulated AXL partly by competing for miR‐7‐5p binding. A, The Western blot analysis showed that the AXL and AKT protein levels increased in over‐linc00852 143B cells. B, The target scan analysis revealed the binding site of linc00852 and miR‐7‐5p and the primer sequence. C, According to qRT‐PCR the expression of miR‐7‐5p in cells with high AXL expression was significantly lower than that in cells with low AXL expression. D, The WT‐linc00852 vector (or Mut‐linc00852) and miR‐7‐5p mimics were co‐transfected into 293T cells; the miR‐7‐5p mimic‐transfected cells reduced the luciferase activity of WT‐linc00852 vector compared with the mutant vector. E, The same results of linc00852 and miR‐7‐5p were further confirmed in 143B cells. F, The WT‐AXL vector (or Mut‐AXL) and miR‐7‐5p mimics were co‐transfected into 293T cells. The miR‐7‐5p mimic reduced the luciferase activities of the AXL WT reporter vector but not the AXL mutant reporter vector. G, The same results of AXL and miR‐7‐5p were further confirmed in 143B cells. (*P < .05, **P < .01, ***P < .001)

By Western blotting, we found that the AXL and AKT protein expression increased significantly in 143B transfected with full linc00852 sequence compared with the control cell (Figure 4A), indicating that linc00852 could increase AXL expression through the AKT pathway. To identify the potential molecular mechanism of linc00852, Starbase (http://starbase.sysu.edu.cn/index.php) was used to investigate the interaction between linc00852 and other transcription factors. According to the predictions, linc00852 could bind to four microRNAs including miR‐7‐5p, miR‐543, miR‐365a‐3p, and miR‐145‐5p. Among them, only miR‐7‐5p was expected to bind to the AXL mRNA 3’UTR. On the other hand, we could not confirm a direct binding site between linc00852 and AXL mRNA by using BLAST (http://blast.ncbi.nlm.nih.gov/Blast.cgi). Interestingly, the analysis by Target Scan showed that linc00852, miR‐7‐5p, and AXL mRNA are correlation in structure and share the same ‘seed’ sequence (Figure 4B). The qRT‐PCR analysis verified that the level of miR‐7‐5p was significantly lower in 143B and HOS cells with high AXL expression than those with low AXL, indicating the negative correlation between miR‐7‐5p and AXL (Figure 4C143B; *P* = .025, HOS; *P* = .013). For further confirmation, dual luciferase reporters including wild‐type (WT) or mutated (Mut) sequences of the miR‐7‐5p binding site in linc00852 were constructed (Figure S11). In 293T cells (Figure 4D,F) and 143B osteosarcoma cells (Figure 4E,G), when miR‐7‐5p mimic was co‐transfected with linc00852‐WT reporter, the luciferase activities reduced significantly. Similar results were obtained when miR‐7‐5p mimic was co‐transfected with AXL‐WT reporter. However, there was no significant change in luciferase activity when the mutant luciferase vector and miR‐7‐5p mimics were co‐transfected. The results suggested that there existed a competing endogenous RNAs (ceRNA) regulatory network among linc00852, AXL mRNA, and miR‐7‐5p in osteosarcoma cells.

### 2.6 Linc00852 might function as a critical transcription factor or a cofactor to facilitate AXL expression in the nucleus

**FIGURE 5:**
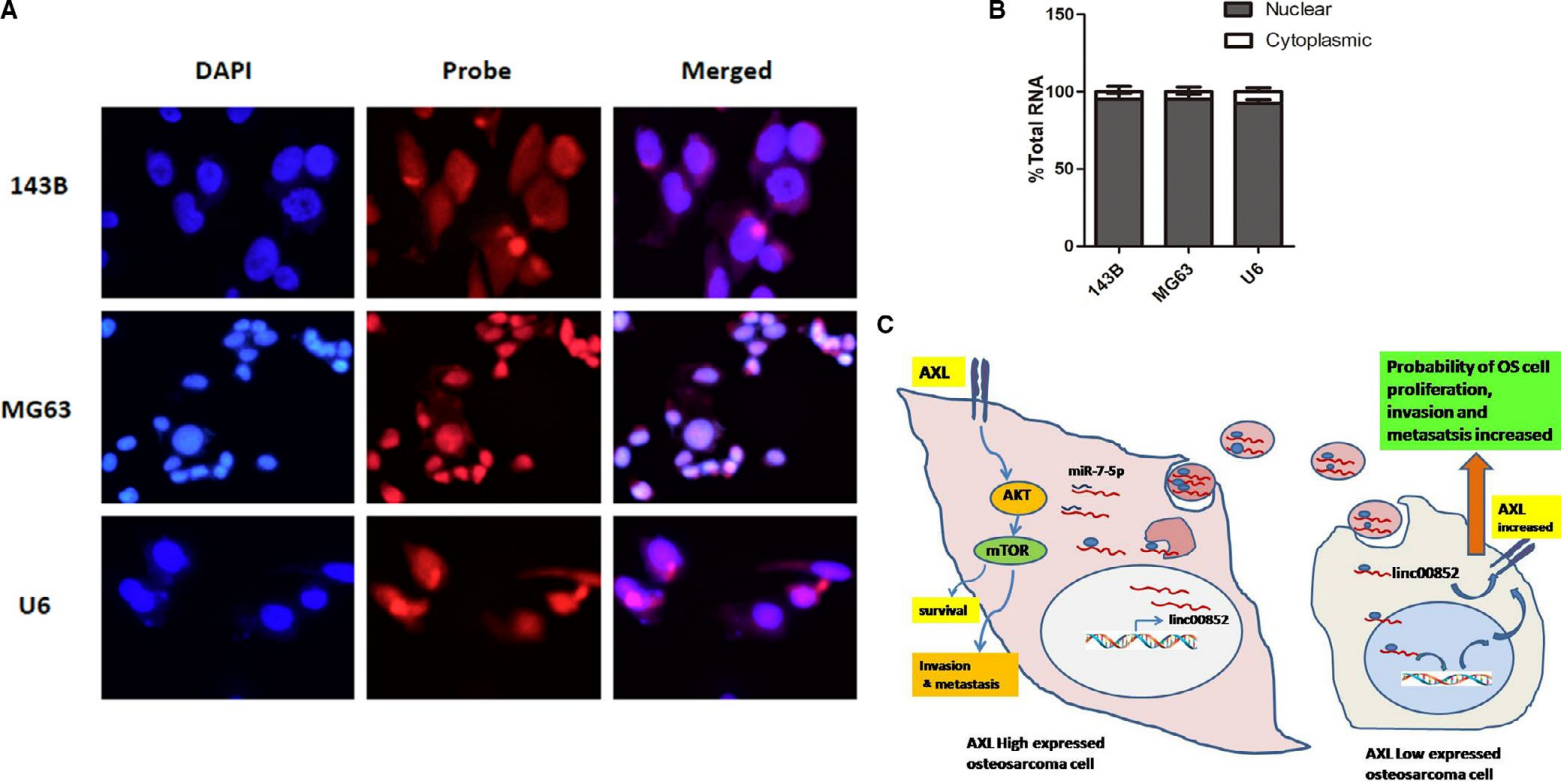
linc00852 located mainly in the nucleus of osteosarcoma cells and the possible mechanism of AXL‐exosome‐linc00852‐AXL positive feedback loop. A, RNA FISH of 143B and HOS cells showed that linc00852 was primarily located in the nucleus, and only a small number was located in the cytoplasm.U6 was used as the nuclear control. B, The qRT‐PCR of cellular fractions further confirmed that linc00852showedintracellular localization. C, Schematic diagram of exosomal linc00852‐mediated osteosarcoma growth and progression. Donor cells with high AXL expression secreted exosomal linc00852; then, the exosomes were transmitted to receiver cells with low AXL expression. A few linc00852 might function as a ceRNA for miR‐7‐5p to facilitate AXL expression in the cytoplasm while most linc00852 might transfer to the receiver cells’ nucleus and increase the expression of AXL with unknown complex mechanisms, sequentially activating the AKT pathway to promote the progression of the receiver cells.

FISH assay in 143B and MG63 demonstrated that linc00852 was mainly located in the nucleus of osteosarcoma cells (Figure 5A). The cellular fractionation assays further confirmed the nuclear enrichment of linc00852 (Figure 5B; *P* = .000). The nuclear accumulation of lncRNAs indicated that there was a potential retention mechanism to prevent the output, additionally with the association of nuclear proteins.^15^, ^16^ Nuclear lncRNAs played an important role in many biological processes, containing chromatin organization, transcriptional and post‐transcriptional gene expression, and serve as structural scaffolds of nuclear domains.^17^ However, the exact regulatory mechanism of nuclear linc00852 is so far unrevealed. In our study, we proved that the donor osteosarcoma cells with high AXL expression could transfer the exosomal linc00852 to the receiver cells with low AXL and increase the abilities of proliferation, migration, and invasion of the receiver cells. Linc00852 increased AXL expression and promoted the progression of osteosarcoma through the AXL‐AKT pathway. Nuclear linc00852 might regulate the AXL gene expression via much more complex molecular mechanisms which needed to be proved in the future (Figure 5C).

### 2.7 Linc00852 increased in osteosarcoma tissues and correlated with tumor progression and the prognosis of the patients

**FIGURE 6:**
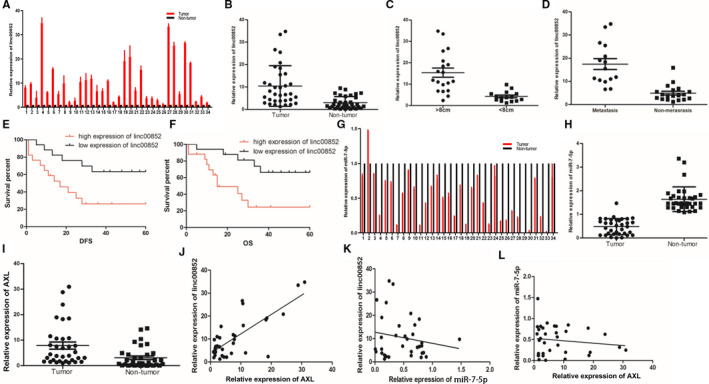
The expression of linc00852, AXL mRNA and miR‐7‐5p in osteosarcoma tissues and the relationship among them. A, The expression of linc00852 in osteosarcoma tissues and adjacent tissues in patients. B, linc00852 levels were significantly higher in osteosarcoma tissues than in the adjacent normal tissues from 34 patients. C, linc00852 expression in tumors with a diameter greater than 8 cm increased significantly versus in the tumors smaller than 8 cm. D, linc00852 expression in tumors with metastasis increased significantly compared with the tumors without metastasis. E, The Kaplan‐Meier analysis revealed that patients with higher linc00852 expression had lower disease‐free survival (DFS). F, Patients with higher linc00852 also had lower overall survival (OS). G, The expression of miR‐7‐5p in osteosarcoma tissues and adjacent tissues in patients. H, miR‐7‐5p levels were significantly lower in osteosarcoma tissues than in the adjacent normal tissues. I, AXL levels were significantly higher in osteosarcoma tissues than in the adjacent normal tissues. J, In the clinical specimens, there was a positive relationship between AXL mRNA and linc00852. K, There was a negative correlation trend with no significance between miR‐7‐5p and linc00852 in osteosarcoma. L, There was a negative correlation trend with no significance between AXL mRNA and miR‐7‐5p in osteosarcoma

According to the qRT‐PCR analysis, the level of linc00852 in osteosarcoma tissues was almost 10 times higher than that in adjacent nontumor tissues of 34 patients (Figure 6A; *P* = .000). According to the clinical features, the tumors could be divided into metastatic and nonmetastatic groups. On the other hand, according to the primary tumor sizes, they were also divided into two groups with large diameter (>8 cm) and with small diameter(<8 cm). The levels of linc00852 were obviously higher in the tumors with metastasis or large diameters than that in the tumors without metastasis or with small diameters (Figure 6B‐D; *P* = .000). In addition, Kaplan‐Meier analysis showed that the patients with linc00852 high expression have lower survival rates of DFS and OS (Figure 6E; *P* = .000, 6F; P = 0.011). Cox's proportional hazards regression demonstrated that highly expressed linc00852 was an independent predictor of the prognosis for the osteosarcoma patients (Table 1). Conversely, the relative level of miR‐7‐5p in osteosarcoma tissues was only half of the nontumor tissues(Figure 6G,H; *P* = .000). qRT‐PCR results of miR‐7‐5p and AXL mRNA in the same clinical specimen showed that AXL was almost seven times higher in tumor tissues (Figure 6I; *P* = .000, Figure S12), and there was a significantly positive association between AXL and linc00852 (Figure 6J; *P* < .001). Although miR‐7‐5p exhibited negative correlation trend between both AXL and linc00852, it were not significant (Figure 6K; *P* = .49, Figure 6L; *P* = .31). Taken together, linc00852 significantly increased in osteosarcoma tissues, and it was positively associated with tumor volume, metastasis, and the expression of AXL mRNA, and it functioned as an oncogene to facilitate osteosarcoma initiation and development.

**TABLE 1:** Multivariable Cox's regression analysis of prognostic factors of the patients

Clinical pathologic characteristics	Overall survival hazard ratio (95% CI)	*P*
Linc00852 expression (high vs low)	0.247(0.084‐0.730)	.011

Note

There was a 95 percent confidence that the overall survival rate of the patients with high expression of linc00852 was only 0.084‐0.73 of those with linc00852 low expression.

## 3 DISCUSSION

Abnormal expression of Gas6/AXL has been confirmed in more and more human malignancies, including pancreatic cancer, breast cancer, NSCLC, glioma, and so on.^4^, ^18^ AXL promotes tumor cell self‐renewal, EMT, and drug resistance, and it has become a novel attractive molecular target for anti‐neoplastic drug discovery.^5^, ^6^, ^19^, ^20^ Studies demonstrated that AXL promoted osteosarcoma progression through activating AKT expression and that it was downregulated by miRNA as miR‐199a and miR‐33a‐5p.^1^, ^7^ We had demonstrated that osteosarcoma could be divided into two tumor groups with different probabilities of invasion and metastasis according to the expression level of AXL. In this study, we found that exosomes, which emerged as novel cell‐cell communication mediators, were transferred from donor cells with high AXL expression to receiver cells with low AXL expression in osteosarcoma to carry the regulative message in the progression of the tumor. Through the high‐throughput sequencing of lncRNA in the exosomes from cells with high and low AXL expression, linc00852 was illustrated as the highest differentially expressed lncRNA and proven to promote the proliferation, invasion, and metastasis of osteosarcoma cells with low AXL in vitro and in vivo. Increased expression of linc00852 caused AXL and AKT overexpression, indicating that linc00852 could promote the progression of osteosarcoma cells through the AXL‐AKT pathway. Thus, we demonstrated a positive feedback loop that regulates exosomal linc00852 and AXL in osteosarcoma. Nevertheless, we still did not know the mechanisms of how exosome‐induced linc00852 dysregulated AXL mRNA and protein.

Many lncRNA mediated gene regulation mechanisms have been identified in the cytoplasm. lncRNAs containing multiple binding sites for the same miRNA are called competing endogenous RNAs (ceRNAs),which can isolate miRNAs and protect its target mRNA from being blocked.^19^, ^21^ As we hypothesized, linc00852 functioned as a ceRNA for miR‐7‐5p to facilitate AXL expression in the cytoplasm. Although linc00852 did not directly bind to AXL mRNA, they shared the same “seed” binding sites as miR‐7‐5p. We also illustrated that overexpression of linc00852 was positively associated with AXL mRNA and showed a negative relationship with miR‐7‐5p, which strongly revealed that a regulatory network existed between these three factors in osteosarcoma. Surprisingly, FISH and the cellular fractionation assays demonstrated that linc00852 was primarily localized in the nucleus. Those results changed the conclusion, which was nearly summarized that exosomal linc00852 could regulate the AXL expression as a ceRNA for miR‐7‐5p.

The human genome encodes nearly 16 000 lncRNAs, most of which are retained in the cell nucleus, these nuclear RNAs are involved in many important physiological and pathological processes. Most nuclear lncRNAs are associated with chromatin and influence gene expression in a cis or trans fashion.^17^ Some lncRNAs affect chromatin organization by binding to proteins, they operate biological functions without directly interacting with chromatin such as BCAR4 in breast cancers.^22^ Nuclear lncRNAs also promotes gene regulation through regulating the association of transcription factors or co‐factors with chromatin as PURPL in colorectal cancer.^23^ Also, there were other researches of nuclear lncRNA such as HERVH, RBM5‐AS1.^24^, ^25^ Until now, only one study has been conducted on linc00852, which promote lung adenocarcinoma progression and spinal metastasis by activating MAPK pathway.^26^

In this study, we have proven that exosomal linc00852 plays as a pivotal identified intercellular messenger in osteosarcoma. Exosomes are not only a cell‐cell communication regulator but also an important mediator in the process of vascular remodeling, playing a mediating role in tumor microenvironment and promoting tumor metastasis.^11^, ^27^, ^28^ Moreover, premetastatic state formation and metastasis relied on cells communication via exosomes between primary tumor cells and distant organ microenvironment.^29^ Exosomes containing miR‐675 have been demonstrated to facilitate tumor cell migration and invasion by targeting CALN1 in metastatic osteosarcoma.^30^ Exosomal lncRNA‐UCA1 was shown to remodel the tumor microenvironment and promote tumor development in bladder cancer cells.^31^ Exosomal lncARSR could be incorporated into exosomes and transmitted to sensitive cells and then promote sunitinib resistance via competitively binding miR‐34/ miR‐449 to facilitate AXL and c‐MET expression in renal cancer cells.^19^ According to the current understanding, exosomes can be applied as diagnostic markers, and exosome‐based drug delivery systems are going to be improved.^32^ In osteosarcoma, several exosome‐derived miRNAs have been shown while exosome‐derived lncRNAs are scarce.^33^, ^34^

In conclusion, our study demonstrated that osteosarcoma cells with high AXL expression promoted growth, invasion and metastasis of the other tumor cells with low AXL expression through releasing linc00852‐containing exosomes and a positive feedback regulation loop between AXL and exosomal linc00852. Exosomal linc00852 may be considered a new tumor biomarker and a special therapeutic target for osteosarcoma.

## 4 MATERIALS AND METHODS

### 4.1 Cell culture

Human osteosarcoma cell lines 143B, HOS, MG63, SaoS2, U2OS were used. All cell lines were grown in DMEM (GIBCO,USA), contained with 10% fetal bovine serum and 100U/ml penicillin/streptomycin solution(GIBCO). Cells were cultured at 37°C in a 5% CO_2_ incubator.

### 4.2 Flow cytometry

The osteosarcoma cell lines were adjusted to (2‐5) × 10^7^/mL and incubated with Alexa Flour 488‐labeled AXL antibody for 1 hour Nonstained cells were used as negative controls. AXL high expression and low expression groups were sorted and recovered by flow cytometry (BD influx).

### 4.3 Extraction and detection of exosomes

The exosomes of osteosarcoma cells were collected, and the morphology of the exosomes was observed by electron microscopy, the exosome‐specific proteins CD63, CD81, TSG101, and Alix were detected according to the supplemental methods.

### 4.4 Exosomal particle size analysis

The exosomes were resuspended with PBS and cooled in an ice bath. The disposable sample reservoir was cleaned with dust‐free paper. The exosomes suspension was injected carefully, and the sample reservoir was sealed and placed in the instrument. The size of the exosomes was detected by Nano series‐Nano‐ZS (Zetasizer).

### 4.5 High‐throughput sequencing

High‐throughput sequencing analyses of exosomal lncRNAs from osteosarcoma cell 143B with high and low AXL expression were performed by Ribobio (Guangzhou) with HiSeq3000 (illumine).

### 4.6 Migration and invasion assays in vitro

The assays were performed according to the methods described in the supplemental methods by osteosarcoma cell lines 143B and HOS with high and low AXL expression, or the cells with low AXL expression cocultured with the exosomes derived from cells with high AXL, or the cells transfected with the lncRNA.

### 4.7 Tumor xenograft models in vivo

Male nude mice (BALB/C Nude, 5‐6 weeks old, 16‐18 g) were maintained in the experimental animal center of SunYat‐Sen University. The animal experiments were performed with the approval of the Animal Ethics Committee of SunYat‐Sen University. Tumor xenograft models were set up according to the methods described in the supplemental methods.

### 4.8 Clone formation assay in vitro

The assays of different groups of vector‐transfected 143B cells were performed according to the methods described in the supplemental methods.

### 4.9 Western blot

The relative proteins as TSG101, Alix, Axl, and Akt (Cell Signaling Technology) were detected.

### 4.10 RNA isolation and quantitative real‐time PCR

According to the methods described in the supplemental methods.

### 4.11 Plasmid construction and lentiviral transfection

The lentiviral vector(GV367) encoding the full linc00852 sequence and the Lenti‐shRNA vector system (GV248, linc00852‐shRNA 1,2,3, and Control shRNA) were constructed by GeneChem (Shanghai) and were operated according to the methods described in the supplemental methods.

### 4.12 RNA fluorescence in situ hybridization (FISH)

RNA probes were synthesized by Ribobio (Guangzhou), and lncRNA was localized in osteosarcoma cell lines 143B and MG63 according to the methods described in the supplemental methods.

### 4.13 Cellular fractionation assay

Cytoplasmic and nuclear RNA separation was performed using the Cytoplasmic and Nuclear RNA Purification Kit (NorgenBiotek). lncRNA was extracted, separated, eluted, and detected by qRT‐PCR in osteosarcoma cell lines 143B and MG63.

### 4.14 Luciferase reporter assay

miR‐7‐5p mimics, control mimics, and pmiR‐RB‐ ReportTM vector containing wild‐type or mutant 3′UTR of AXL and linc00852 were constructed by Ribobio(Guangzhou). The 293T cells and 143B osteosarcoma were used according to the methods described in the supplemental methods.

### 4.15 Patients and specimens

Fresh primary tumor tissues and paired adjacent nontumor soft tissues from 34 patients with osteosarcoma(Table S1) undergoing surgical resection at the First Affiliated Hospital of SunYat‐Sen University from 2011 to 2014 with the details in the supplemental methods.

### 4.16 Statistical analysis

All statistical analyses in this study were performed using SPSS 20.0 software. Student's *t* test was used to analyze the significance of mean values between the two groups. Spearman's and Pearson's correlations were performed to determine the correlation between two variables. Survival curves were constructed using the Kaplan‐Meier's method and calculated by a log‐rank test. A Cox proportional hazards regression analysis was used to analyze the effect of clinical variables on patient survival. A *P* value < .05 was considered significant.

## CONFLICT OF INTEREST

It has been submitted with the full knowledge and approval of the First Affiliated Hospital, Sun Yat‐sen University. All authors have significantly contributed and are in agreement with the content of the final manuscript. The authors declare that there are no conflict of interest in connection with the work submitted.

## AUTHOR CONTRIBUTIONS

TP designed the experimental objective and made the final decision for the manuscript. QL and XW completed the main experiments and drafted the manuscript and literature review. NJ performed the figures and helped to check and refine the manuscript. XX collected the clinical data. NL carried out morphological experiments. JL assisted with the xenograft animal experiments and JS helped provide clinical information.

## Supporting information

Fig S1

Fig S2

Fig S3

Fig S4

Fig S5

Fig S6

Fig S7

Fig S8

Fig S9

Fig S10

Fig S11

Fig S12

Supplementary Material

## Data Availability

The data that support the findings of this study are available from the corresponding author upon reasonable request.
