# Supplementary figures and images for: Exosome‐transmitted linc00852 associated with receptor tyrosine kinase AXL dysregulates the proliferation and invasion of osteosarcoma
Source: Cancer Med. 2020 Jul 16;9(17):6354–66. doi: 10.1002/cam4.3303 (PMC7476833; doi:10.1002/cam4.3303)

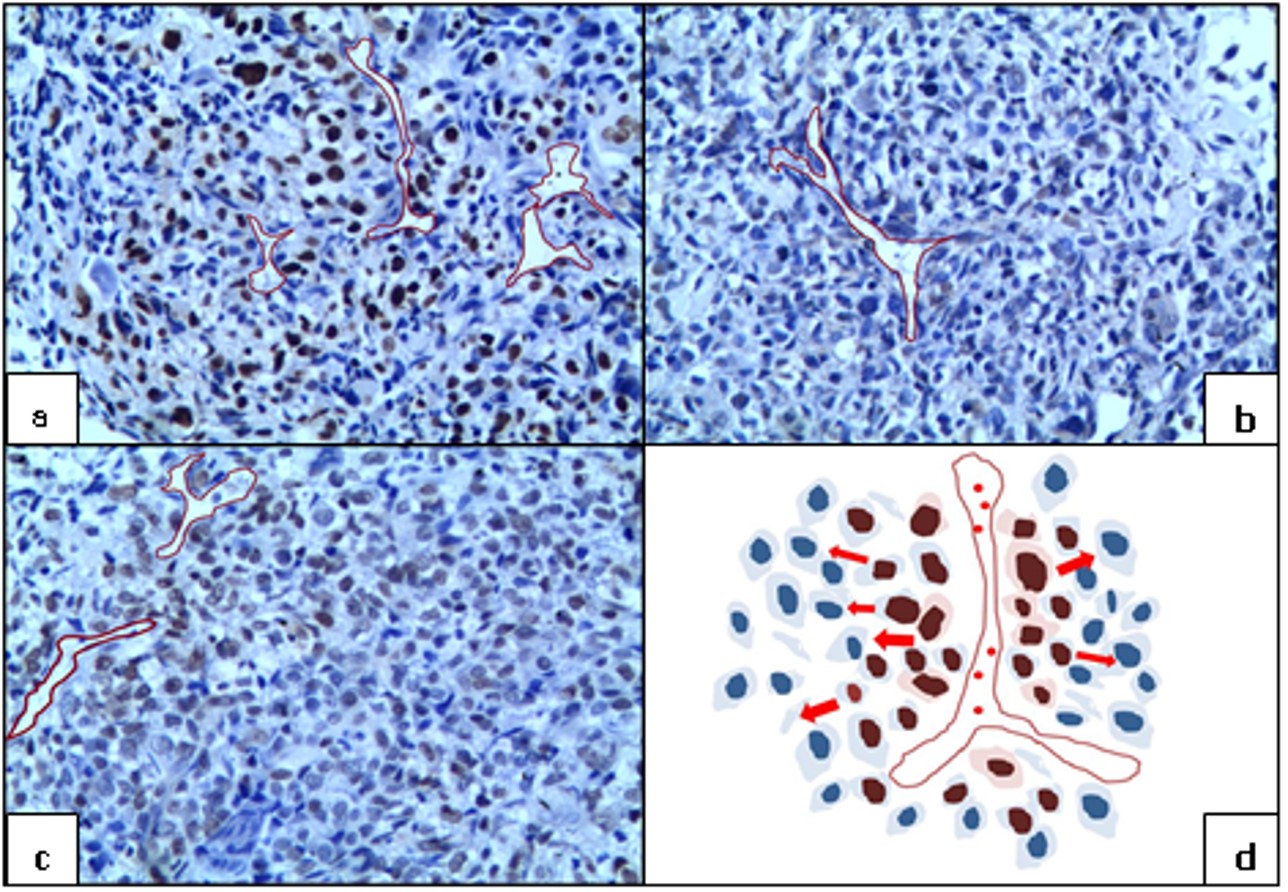

Supplement: Supplementary file 1 — Fig S1 [file CAM4-9-6354-s001.jpg]

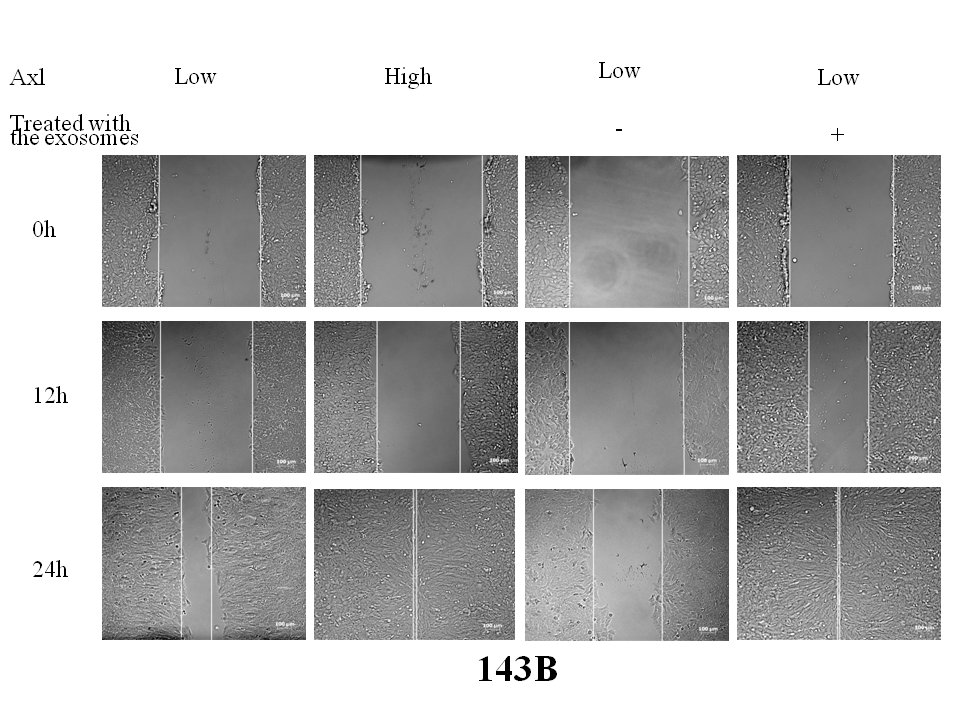

Supplement: Supplementary file 2 — Fig S2 [file CAM4-9-6354-s002.tif]

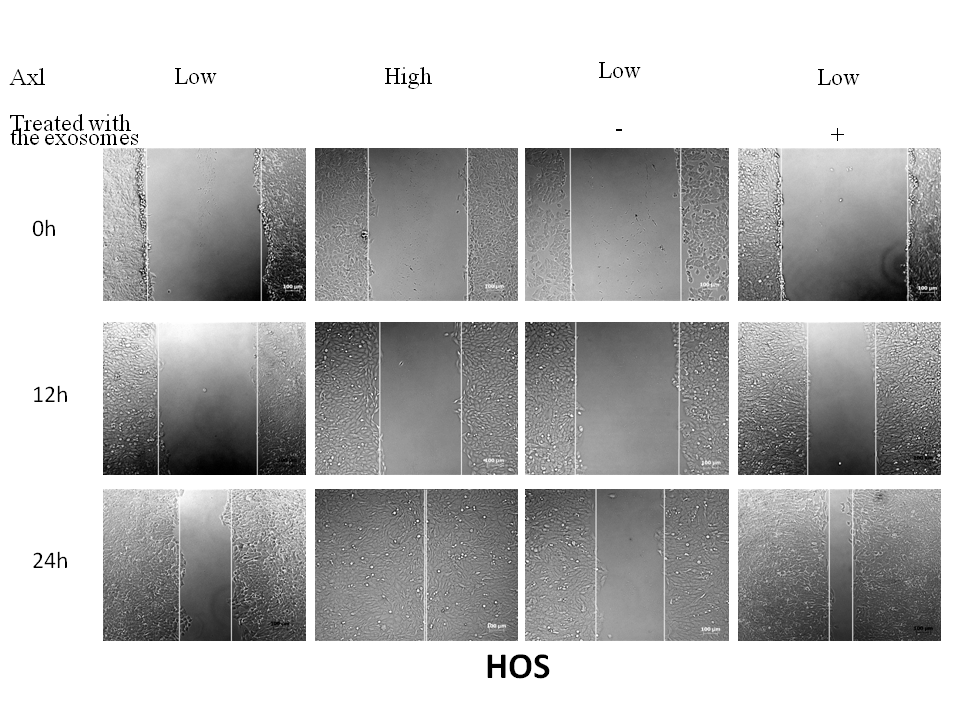

Supplement: Supplementary file 3 — Fig S3 [file CAM4-9-6354-s003.tif]

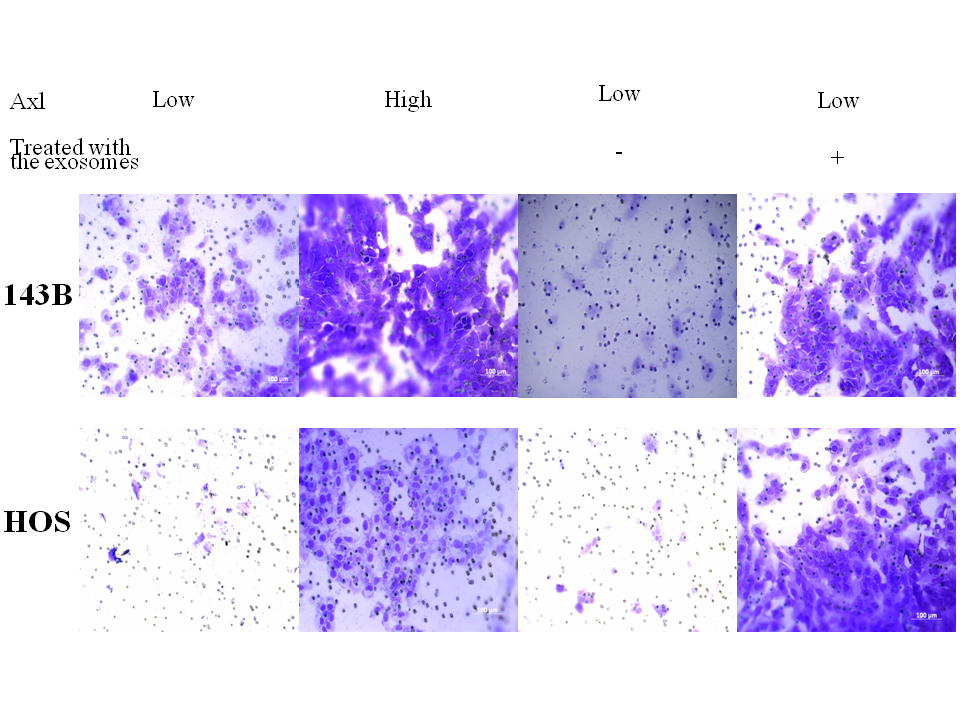

Supplement: Supplementary file 4 — Fig S4 [file CAM4-9-6354-s004.tif]

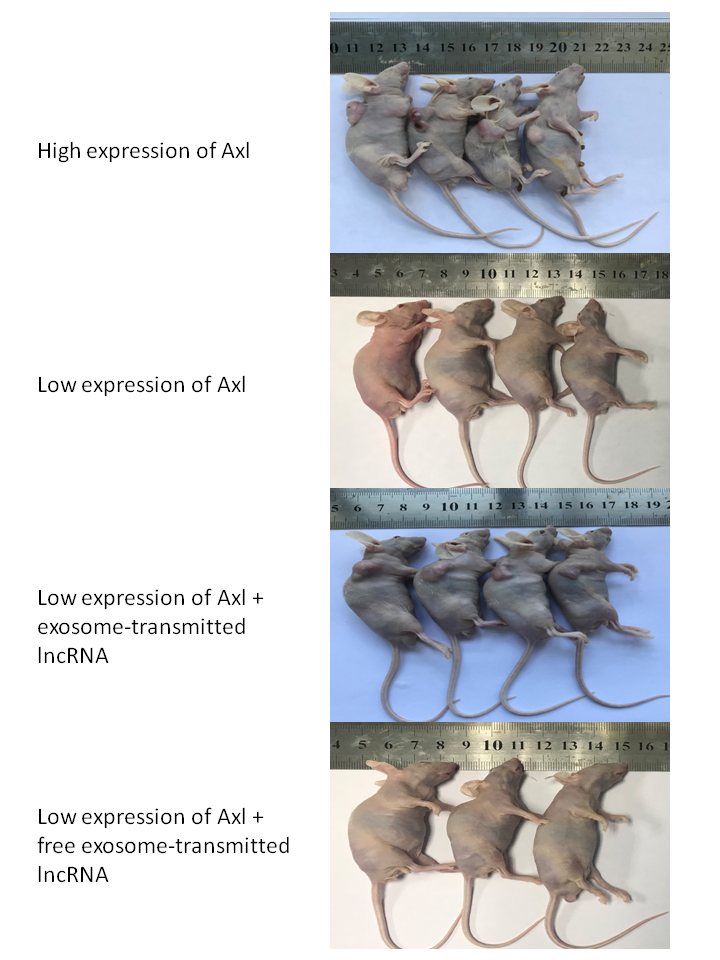

Supplement: Supplementary file 5 — Fig S5 [file CAM4-9-6354-s005.tif]

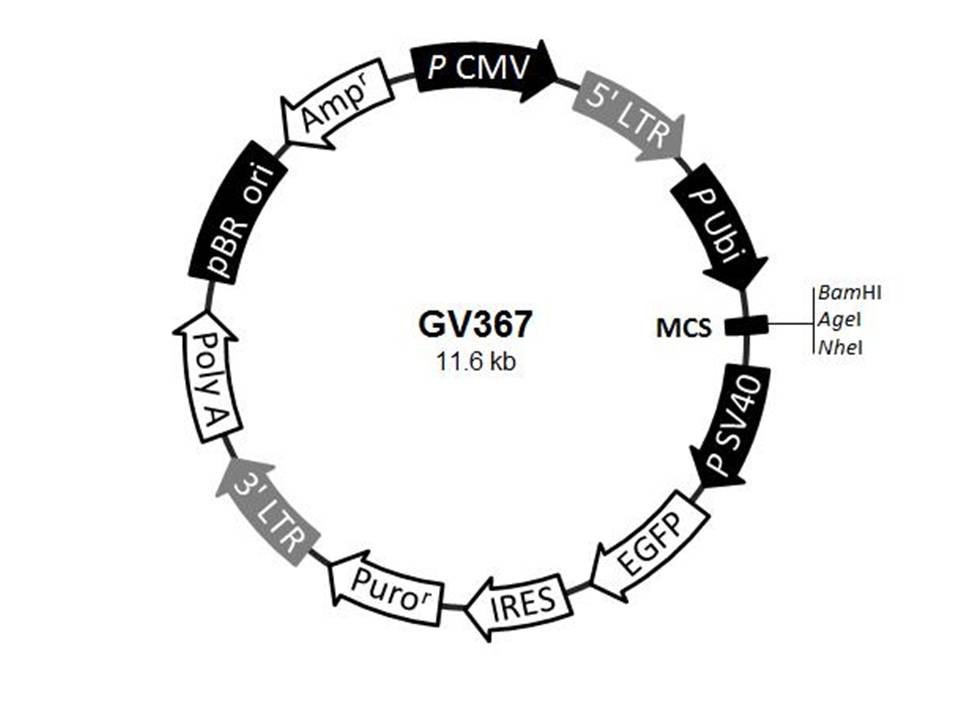

Supplement: Supplementary file 6 — Fig S6 [file CAM4-9-6354-s006.jpg]

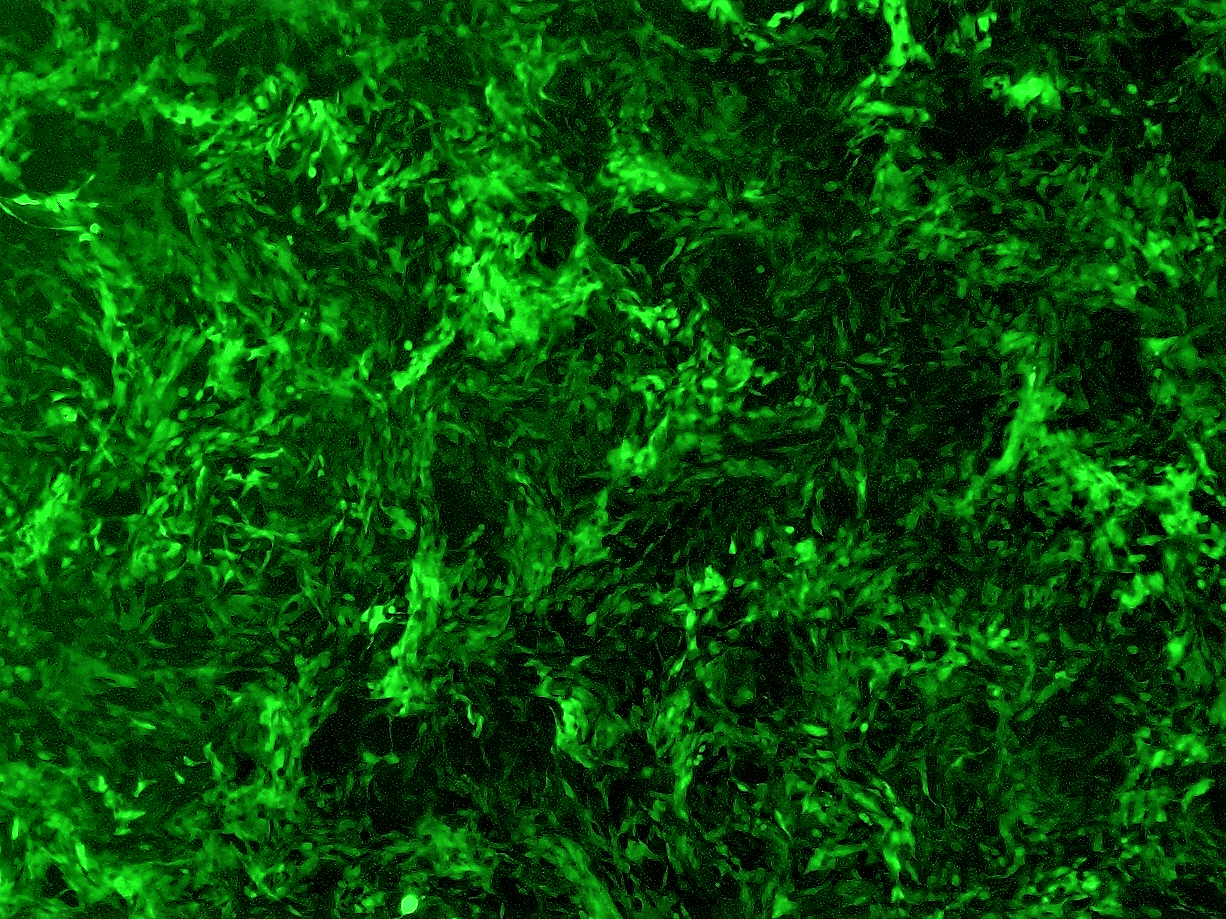

Supplement: Supplementary file 7 — Fig S7 [file CAM4-9-6354-s007.tif]

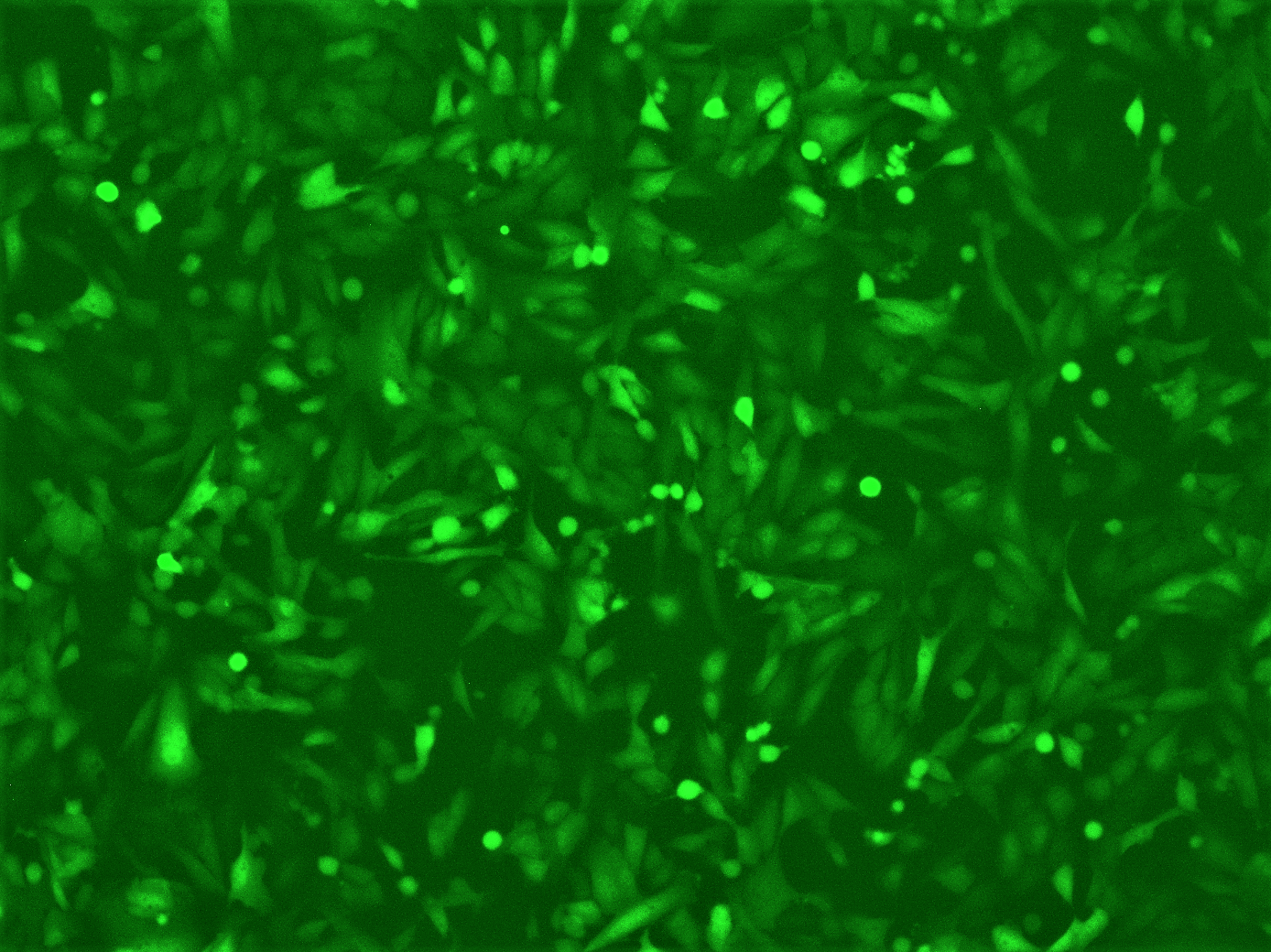

Supplement: Supplementary file 8 — Fig S8 [file CAM4-9-6354-s008.tif]

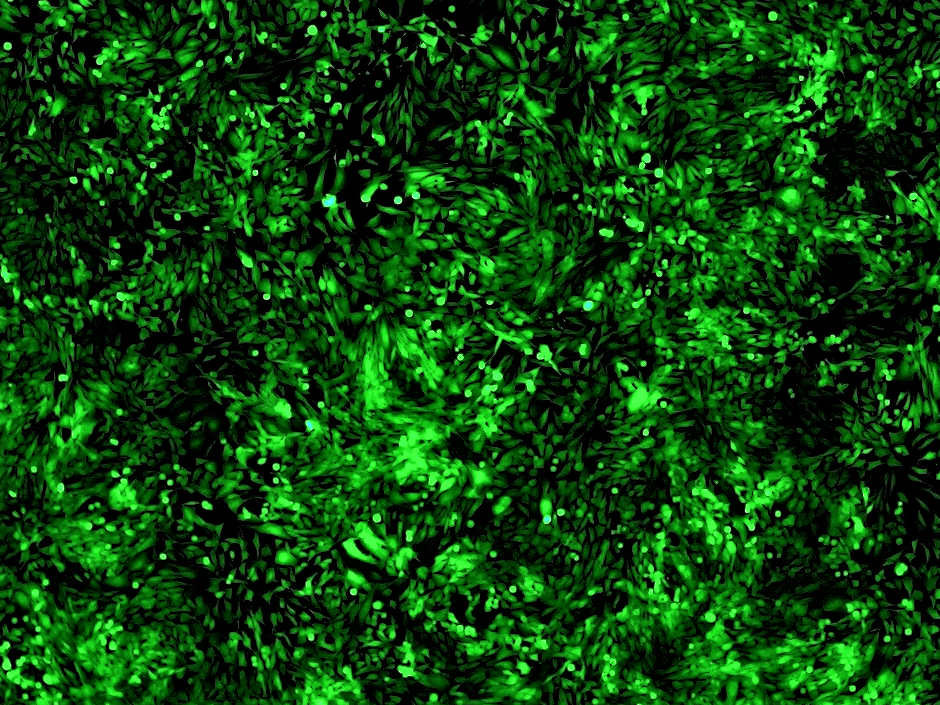

Supplement: Supplementary file 9 — Fig S9 [file CAM4-9-6354-s009.tif]

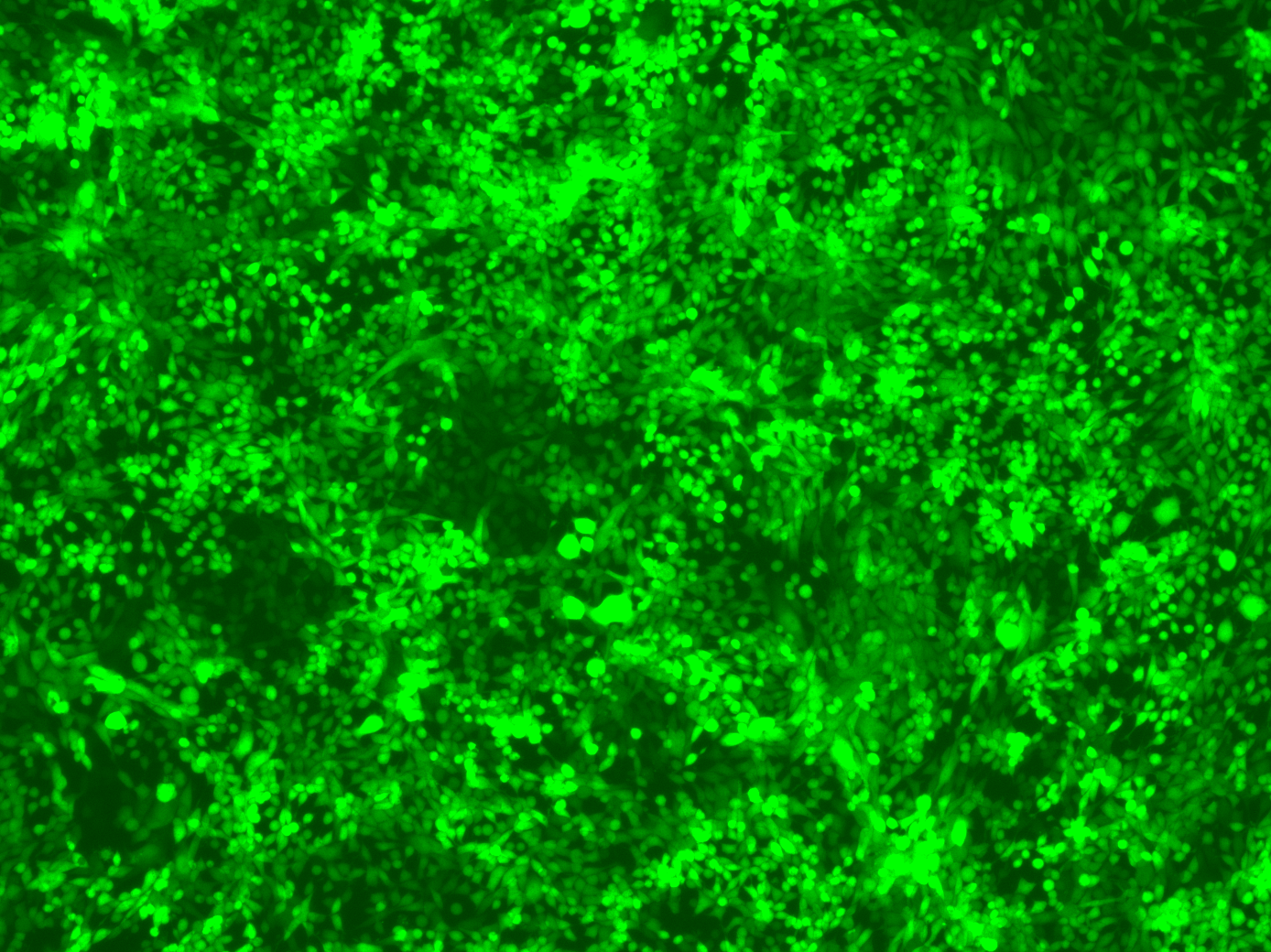

Supplement: Supplementary file 10 — Fig S10 [file CAM4-9-6354-s010.TIF]

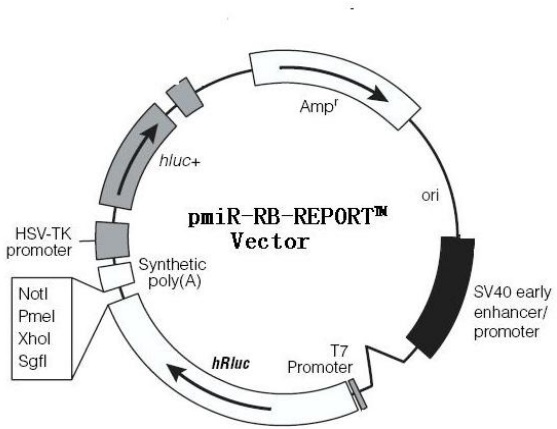

Supplement: Supplementary file 11 — Fig S11 [file CAM4-9-6354-s011.jpg]

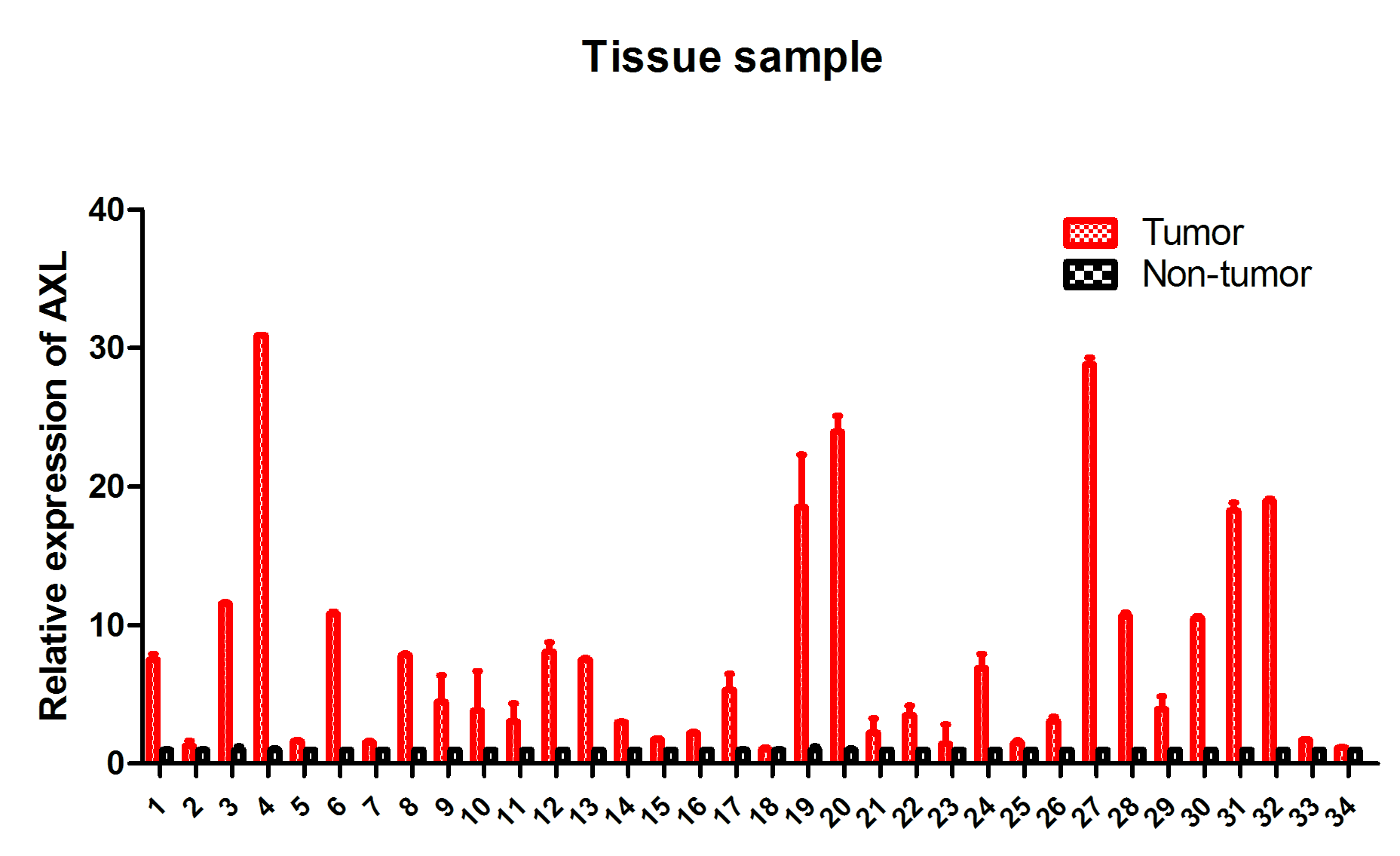

Supplement: Supplementary file 12 — Fig S12 [file CAM4-9-6354-s012.tif]
