# Supplementary material for: Exosome‐transmitted linc00852 associated with receptor tyrosine kinase AXL dysregulates the proliferation and invasion of osteosarcoma
Source: Cancer Med. 2020 Jul 16;9(17):6354–66. doi: 10.1002/cam4.3303 (PMC7476833; doi:10.1002/cam4.3303)
Supplement: Supplementary file 13 — Supplementary Material [file CAM4-9-6354-s013.docx]

**Supplemental Materials and Methods**

**Extraction and detection of exosomes.** The osteosarcoma cells were grown in a serum-free medium. After 72 hours the conditioned medium was collected, the cells and cell debris were removed by differential centrifugation, and the supernatant was filtered through a 0.2 μm membrane. Then, the exosomes from the supernatant were isolated by 120min ultracentrifugation at 100,000g, 4°C. The morphology of the exosomes was observed by electron microscopy, the exosome-specific membrane proteins CD63 and CD81were detected by flow cytometry, and the specific markers TSG101 and Alix of exosomes were detected by Western blotting.

**Migration and invasion assays in vitro.** Wound healing assays were performed to examine the migration ability of the 143B and HOS osteosarcoma cell lines. Pictures were acquired with a Leica DMI4000B microscope (Leica, Germany). Invasion assays were performed in 8 μm pore size transwell chambers (Corning, USA) and a Boyden chamber covered with Matrigel matrix (Corning, USA). A total of 200µl transfected cells or exosome-treated osteosarcoma cell HOS (2×10^5^/ml) or 143B (1×10^5^/ml) cell suspensions from different groups were seeded in the upper chambers pre-coated with Matrigel™ Matrix (BD Biosciences, CA, USA) and cultured in serum-free basal medium. A total of 500µl medium containing 10% FBS was added to the lower chambers. After 24 h, cells that had invaded to the bottom surface were fixed with 4% paraformaldehyde and stained with 1% crystal violet. The invading cells were counted and averaged from five randomly selected fields.

**Tumor xenograft models in vivo.** Male nude mice (BALB/C Nude, 5-6 weeks old, 16-18g) were maintained in a pathogen-free condition in the experimental animal center of Sun Yat-sen University. The animal experiments were performed with the approval of the Animal Ethics Committee of Sun Yat-sen University. Subcutaneous xenoplastic transplantation was performed by injecting into the mice with 5×10^6^ untreated 143B cells or treated osteosarcoma cells. After 18 days, the mice were euthanized and the tumor volume and weight were measured. Xenograft tumors and the lungs and livers were stained by H&E to find the pathological features and the metastatic foci.

**Clone formation assay in vitro.** Different groups of vector-transfected 143B cells (500 cells/well) in log-phase growth were seeded in 6-well plates and cultured for about 8 days, then treated with 4% paraformaldehyde and stained with 0.1% crystal violet. After washing, the plates were air dried and clones containing >50 cells were counted.

**Western blot.** The total proteins were isolated; following measurement of the protein concentration, they were measured and separated by SDS-PAGE and transferred onto a nitrocellulose membrane. The membranes were incubated with TSG101, Alix, Axl, and Akt (Cell Signaling Technology,USA) primary antibodies, and antibodies against GAPDH were used as a loading control. The protein expression was assessed by a chemiluminescent imaging system.

**Plasmid construction and stable cell line transfection.** The lentiviral vector (GV367) encoding the full LINC00852 sequence and the Lenti-shRNA vector system (GV248, linc00852-shRNA 1,2,3, and Control shRNA) were synthesized by ShangHai GeneChem Company (Shanghai, China). The shRNA sequences were listed in TableS1. These products were transformed into Escherichia coli cells and selected for puromycin resistance. The correct vectors were amplified and cotransfected with helper vectors into 293T cells. Infectious lentiviruses were harvested 48 hours post-transfection and filtered through 0.45μmPVDF filters. Recombinant lentiviruses were concentrated by ultra-centrifugation (2h at 25,000×g). After physical state test, sterility test and virus titer test, the lentivirus were dissolved in PBS and stored at -80℃. The 143B and HOS osteosarcoma cells in logarithmic growth phase were seeded into a 24-well plate with about 5×10^4^ cells per well.After attachment, the culture medium was discarded and replaced with a virus infection. After 12 hours, the infection solution was replaced with complete culture media. After 48 hours, The puromycin was added and the transfected cells were obtained. Empty vector and scramble vector containing GFP were used as a negative control. LINC00852 expression was detected by qRT-PCR 96 hours after infection.

**RNA isolation and quantitative real-time PCR.** The total RNA was isolated using Trizol reagent (Invitrogen), and the cDNA was converted from total RNA by using PrimeScript RT Master Mix(TaKaRa,RR036A) according to the manufacturer’s instructions. Quantitative real-time PCR was performed with SYBR Premix Ex Taq II (Takara, RR820A) and detected on the Applied Biosystems®QuantStudio7 (Thermo Fisher Scientific). PCR reaction conditions were set according to the kit protocol. The primers were synthesized by Sangon Biotech(Shanghai, China) and Ribobio(Guangzhou, China) , which were listed in TableS2. The relative expression level of the indicated genes was compared with that of GAPDH, and the expression fold changes were calculated using the 2-ΔCt method. Each sample was assayed in triplicate.

**RNA Fluorescence in situ hybridization (FISH).** FISH experiments were performed according to the manufacturer’s instructions on cell slides in 24-well culture plates (F ISH Kit, Ribobio, China). Briefly, cells were washed and fixed when they reached 60% confluence. After permeabilization, cells were incubated with prehybridization solution at 37°C for 30 minutes. The cells were then incubated with hybridization solution containing LINC00852 probe, U6 or 18S probe (Ribobio, China). After DAPI staining, washing and mounting, images were obtained with a Laser scanning confocal microscope (LSM780, Zeiss, Germany).

**Luciferase reporter assay.** Four pmiR-RB-REPORT™ constructs (Figure S11) containing the AXL 3’-UTR with the putative miR-7-5p binding site, AXL 3’-UTR with a mutant binding site, full LINC00852 sequence with the putative miR-7-5p binding site and full LINC00852 sequence with a mutant binding site were synthesized (Ribobio, China). All constructs were verified by sequencing (SBO, China). 293T cells were plated in 96-well plates and cotransfected with 100ng of the constructs (AXL Mut-3’-UTR, AXL WT-3’-UTR, LINC00852-WT, or LINC00852-Mut) and miR-7-5p mimics or mimic control (50nM/well) by using the riboFECT™ CP Reagent (0.75 µl/well) (Ribobio, China). Mimic control and AXL mut-3’-UTR were used as negative controls. After 24 hours, Firefly and Renilla luciferase activities were measured sequentially by using the dual luciferase assay kit (Promega, USA) following the specification on Tecan Infinite F500 (Tecan Systems,USA). Firefly luciferase activities were normalized with those of Renilla luciferase and expressed as relative Rluc/Luc ratios.

**Patients and specimens.** Fresh primary osteosarcoma samples and paired adjacent non-tumor soft tissues from 34 patients (Table S1) undergoing surgical resection at the First Affiliated Hospital of SunYat-Sen University from 2011 to 2014 were frozen in liquid nitrogen and stored at -80°C for RNA extraction. This study was approved by the Institutional Research Ethics Committee of the First Affiliated Hospital of SunYat-Sen University. Written informed consent was obtained from patients or patients’ families. Follow-up was performed every 2-3 months during the first year after surgery until January 2016. Disease-free survival (DFS) was calculated from the date of tumor resection until the detection of tumor recurrence or metastasis, death, or the last observation. Overall survival (OS) was defined as the length of time between surgery and death or the last follow-up examination.

TableS1. Lentiviral-shRNA forlinc00852 vector system

| shRNA | Target sequence |
| --- | --- |
| SH1 | AAGCGCTCAGATGGCTGAATT |
| SH2 | GCCCTGGCTGGTTGTTAATAA |
| SH3 | GCCCATGTGAGGCATTTGCTT |
| Scramble control | TTCTCCGAACGTGTCACGT |

TableS2. PCR primers

| Linc00852 | Forward | TGGGCACCTCACTTAACGTC |
| --- | --- | --- |
|  | Reverse | TGCAAGGCTGCATTAACAGC |
| AXL | Forward | TCAAGGTGGCTGTGAAGACGA |
|  | Reverse | CGTTCAGAACCCTGGAAACAGAC |
| GAPDH | Forward | GCACCGTCAAGGCTGAGAAC |
|  | Reverse | TGGTGAAGACGCCAGTGGA |
| miR-7-5p | primers | TGGAAGACTAGTGATTTTGTTGT |
